# Post-translational allosteric activation of the P2X_7_ receptor through glycosaminoglycan chains of CD44 proteoglycans

**DOI:** 10.1038/cddiscovery.2015.5

**Published:** 2015-10-05

**Authors:** GEDD Moura, SV Lucena, MA Lima, FD Nascimento, TF Gesteira, HB Nader, EJ Paredes-Gamero, ILS Tersariol

**Affiliations:** 1 Departamento de Bioquímica, Universidade Federal de São Paulo, São Paulo, Brazil; 2 Grupo de Pesquisa em Biomateriais e Biotecnologia, Universidade Bandeirante de São Paulo, São Paulo, Brazil; 3 Division of Developmental Biology, Cincinnati Children's Hospital and Research, Cincinnati, OH, USA; 4 Centro Interdisciplinar de Investigação Bioquıí́mica, Universidade de Mogi das Cruzes, São Paulo, Brazil

## Abstract

Here, we present evidence for the positive allosteric modulation of the P2X_7_ receptor through glycosaminoglycans (GAGs) in CHO (cell line derived from the ovary of the Chinese hamster) cells. The marked potentiation of P2X_7_ activity through GAGs in the presence of non-saturating agonists concentrations was evident with the endogenous expression of the receptor in CHO cells. The presence of GAGs on the surface of CHO cells greatly increased the sensitivity to adenosine 5′-triphosphate and changed the main P2X_7_ receptor kinetic parameters EC_50_, Hill coefficient and *E*_max_. GAGs decreased the allosteric inhibition of P2X_7_ receptor through Mg^2+^. GAGs activated P2X_7_ receptor-mediated cytoplasmic Ca^2+^ influx and pore formation. Consequently, wild-type CHO-K1 cells were 2.5-fold more sensitive to cell death induced through P2X_7_ agonists than mutant CHO-745 cells defective in GAGs biosynthesis. In the present study, we provide the first evidence that the P2X_7_ receptor interacts with CD44 on the CHO-K1 cell surface. Thus, these data demonstrated that GAGs positively modulate the P2X_7_ receptor, and sCD44 is a part of a regulatory positive feedback loop linking P2X_7_ receptor activation for the intracellular response mediated through P2X_7_ receptor stimulation.

## INTRODUCTION

Classical pharmacology postulates that receptor activation occurs through drug-receptor interactions.^1,2^ Advances achieved during the last years have increased the knowledge concerning this activation process and introduced new aspects that might modulate drug-receptor interactions, for example, receptor states, oligomerization with other proteins and allosteric mechanisms.^3^

Extracellular matrix components, such as glycosaminoglycans (GAGs) and proteoglycans have shown the modulation of receptor activity.^4,5^ The action of heparin on the muscarinic receptor M_3_ occurred through the heparin/extracellular matrix molecules/integrin complex, which positively modulates the M_3_ receptor, inducing relaxation through increased nitric oxide levels.^6^ In addition, heparin activates the serotonin receptor and serotonin signaling pathways.^7^ Versican, a cell membrane proteoglycan, promotes the sensitization of mechanosensitive currents in nociceptive sensory neurons.^8^ The interaction between toll-like receptors and biglycan, a leucine-rich repeat proteoglycan, has also been described on macrophages.^9^ Heparan sulfate associated with extracellular matrix components might interact with glutamate receptors, and this interaction could affect the functional properties of these proteins.^10^ The type III transforming growth factor-*β* (TGF-*β*) receptor interacts with heparan sulfate proteoglycan and other GAG chains.^11^

The purinergic P2 receptor family comprises G-protein-coupled receptors (P2Y) and adenosine 5′-triphosphate (ATP)-gated ion channel receptors (P2X). These receptor families have acquired increasing relevance as they comprise 15 members, associated with several biological processes, including muscle contraction, neurotransmission, exocrine and endocrine secretion, immune response, platelet aggregation, inflammation, pain, proliferation, differentiation and cell death.^12–15^

The P2X receptors include seven members (P2X_1-7_) that are activated through ATP or the ATP analogs *αβ*-MeATP (*α,β*-methyleneadenosine 5′-triphosphate), *βγ*-MeATP (*β,γ*-methyleneadenosine 5′-triphosphate) and BzATP (2′(3′)-O-(4-benzoylbenzoyl)adenosine 5′-triphosphate) to promote the opening of ion channels permeable to small cations, such as Na^+^, K^+^ and Ca^2+^, eliciting intracellular signaling responses.^16^ Among them, the P2X_7_ receptor exhibits a different pharmacological feature, prolonged stimulation at the mM ATP range leading to the formation of a nonselective pore in the cellular membrane, which increase the permeability to organic cations of a molecular mass up to 1 kDa.^17,18^ Several studies have associated the opening of this nonselective pore with cytoplasmic calcium influx and membrane depolarization, causing marked physiological impacts that lead to necrosis and apoptosis.^15,19^

The binding of agonists to the P2X_7_ receptor is tightly regulated through allosteric mechanisms, which act on either the extracellular or intracellular/transmembrane domains of the receptor subunits, such as divalent cations, alcohols, metals and pH.^20^ Post-translational mechanisms to regulation P2X receptor activity have also been described, such as phosphoinositides,^21^ phosphorylation^22^ and integrins.^23,24^ Although previous studies have shown that P2X receptors are associated with the regulation of cellular biosynthesis and secretion of proteoglycans,^7^ the influence of GAGs on the physiology of these receptor classes has not been fully evaluated.

Herein we investigated the role of GAGs in P2X_7_ receptor activation. We observed that the presence of GAGs positively regulates the activation of the P2X_7_ receptor; thereby, increasing Ca^2+^ entry and P2X_7_-dependent cell death. This work provided the first evidence that cell surface GAGs have a relevant functional impact on P2X_7_ receptor physiology, emerging as a new cofactor necessary for full receptor activity and a new regulatory mechanism for purinergic signaling at the cellular level.

## Results

### P2 receptor-mediated calcium influx in CHO cells

**Figure 1:**
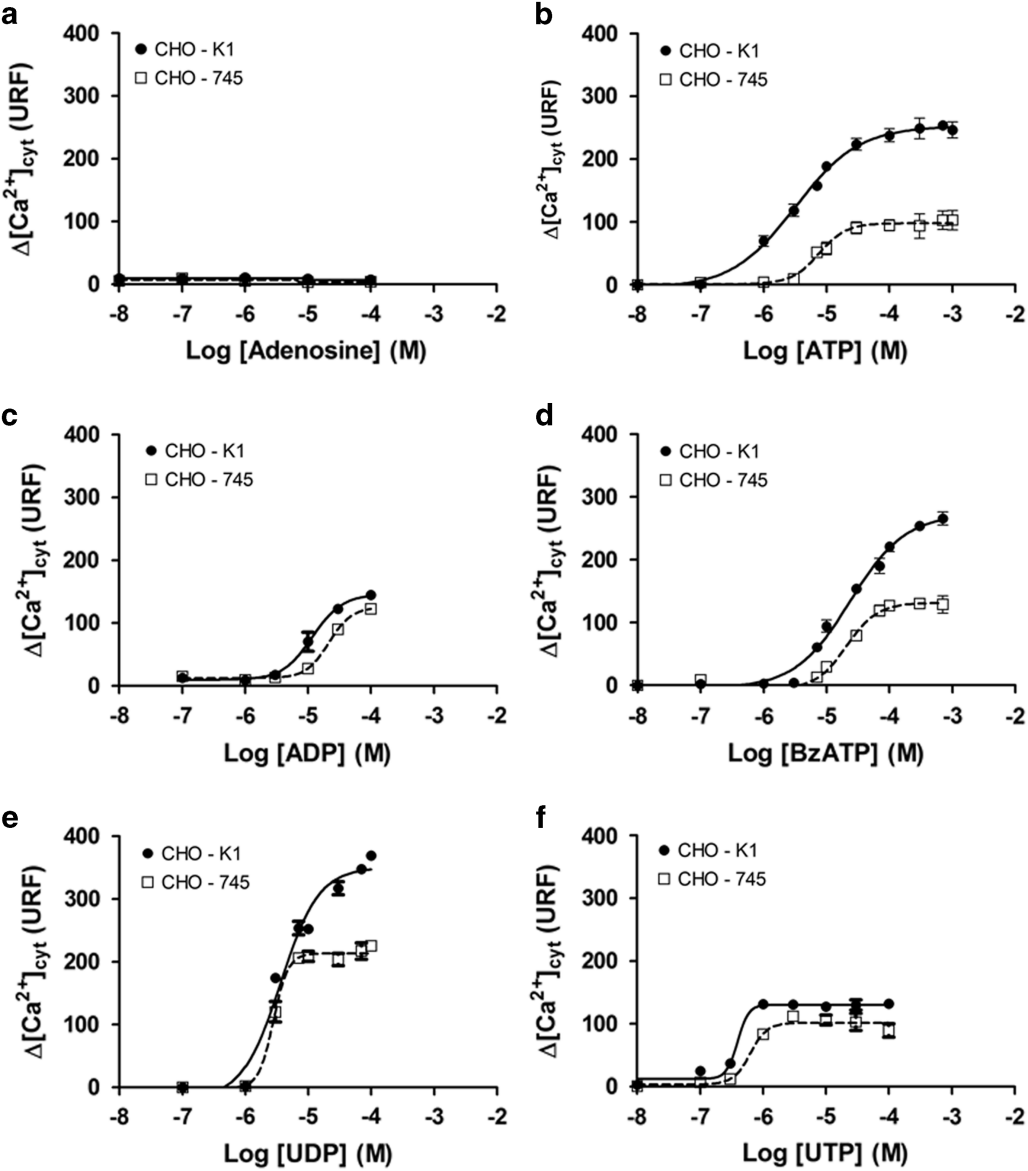
Concentration–response curves for the ability of adenosine, ATP, ADP, BzATP, UDP or UTP to stimulate Ca^2+^ influx to cytoplasm of the CHO cells. Cytoplasmic Ca^2+^ influx, [Ca^2+^]_cyt_, measurements were monitored through changes of the Fluo-4 fluorescence intensity in real time using the Flex Station 3 microplate reader system. CHO-K1 (●-●) and CHO-745 (□-□) cells were seeded onto black 96-well plates (10^4^ cells/well). Then, the cells were incubated with Fluo-4 for 1 h at 37 °C. The samples were monitored for 200 s, and the amplitude of the basal and maximum fluorescence value, after agonist stimulus with adenosine (**a**), ATP (**b**), ADP (**c**), BzATP (**d**), UDP (**e**) or UTP (**f**) to obtain the corresponding concentration–response curves. The data represent the mean±S.E.M. (*N*=6).

To verify whether GAGs modulates the response of P2 receptors, the P2 response to agonists was compared using wild-type CHO-K1 cells and mutant CHO-745 cells, which are defective in GAGs chains. Because most of the P2 receptor activation involves increased Ca^2+^ signaling, concentration–response curves were obtained using different P2 agonists. Adenosine, an ATP degradation product, which actives P1 receptors, was used to exclude the participation of these receptors (Figure 1a). As observed in Figure 1, ATP, ADP (adenosine 5′-diphosphate), UTP (uridine 5′-triphosphate), UDP (uridine 5′-diphosphate) and BzATP promoted Ca^2+^ increase in CHO (cell line derived from the ovary of the Chinese hamster) cells, suggesting that several P2 receptors are expressed in CHO cells. The addition of 2-MeSATP (2-(methylthio)adenosine 5′-triphosphate), *αβ*-MeATP and *βγ*-MeATP did not increase intracellular Ca^2+^, suggesting that the receptors P2X_1_, P2X_2_, P2X_3_, P2X_4_ and P2X_5_ are unlikely involved, consistent with the results of a previous study.^25^ Comparative evaluation of the agonists concentration–response curves showed differences in *E*_max_ when the cells where stimulated with ATP, BzATP and UDP (Figures 1d and e). Typical time courses are shown in Supplementary Figure SI1.

### Characterization of the P2X_7_ receptor response in CHO cells

**Figure 2:**
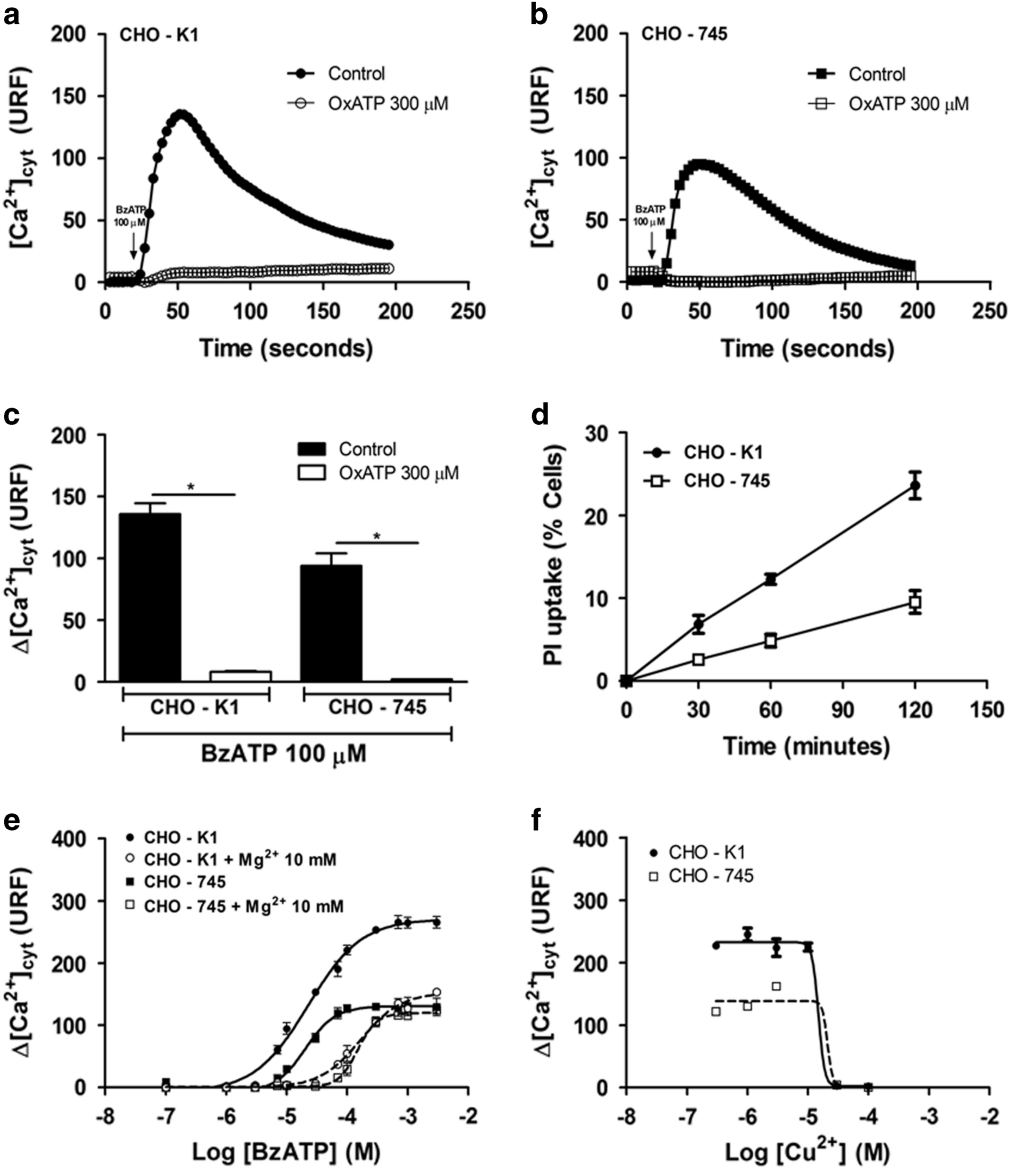
Characterization of the P2X_7_ receptor in CHO cells. Cytoplasmic Ca^2+^ influx, [Ca^2+^]_cyt_, measurements were monitored through changes in the Fluo-4 fluorescence intensity in real time using the Flex Station 3 microplate reader system. Time course curves of the Fluo-4 fluorescence intensity, referent to transient Ca^2+^ influx in CHO-K1 (**a**) and CHO-745 (**b**) cells after stimulation with 100 *μ*M BzATP, in the absence (black symbols) or presence of 300 *μ*M oxATP (white symbols). The cytoplasmic Ca^2+^ increase in the absence (black bars) or presence of oxATP (white bars) in CHO cells is shown in **c**. Time course curves of PI accumulation in CHO-K1 (●-●) and CHO-745 (□-□) cells in response to 4 mM ATP (**d**). Concentration–response curves for the ability of BzATP to stimulate Ca^2+^ influx in CHO-K1 (circles) and CHO-745 (squares) cells in the absence (black) or presence of 10 mM MgCl_2_ (white) (**e**). Concentration–response curves for the ability of Cu^2+^ to block Ca^2+^ influx mediated through 100 *μ*M BzATP in CHO-K1 (●-●) and CHO-745 (□-□) cells (**f**). The data represent the mean±S.E.M. (*N*=6). *P<0.05.

To confirm the presence of the P2X_7_ receptor and compare the differences in the responses between CHO-K1 and CHO-745 cells, pharmacological assays were performed using the antagonist ATP periodate oxidized (oxATP), a selective antagonist of the P2X_7_ receptor.^26^ oxATP abolished the effect of BzATP in both CHO-K1 (Figures 2a and c) and CHO-745 cells (Figures 2b and c). In addition, 4 mM ATP induced the time-dependent uptake of propidium iodide (PI) in CHO cells after 30 min at 37 °C (Figure 2d). Notably, mutant CHO-745 cells were 2.5-fold less sensitive to PI accumulation (Figure 2d). This delayed PI accumulation reflects the slow formation of the putative pore form of the P2X_7_ receptor (Figure 2d), and might suggest that the initial Ca^2+^ influx at 50 s (Figures 2a and b) is the precise channel for the P2X_7_ receptor. Moreover, BzATP-stimulated Ca^2+^ influx is negatively modulated through a high concentration of divalent cations Mg^2+^ (Figure 2e) and Cu^2+^ (Figure 2f).

### Both CHO cells lines did not show differences in the expression and cellular localization of the P2X_7_ receptor

**Figure 3:**
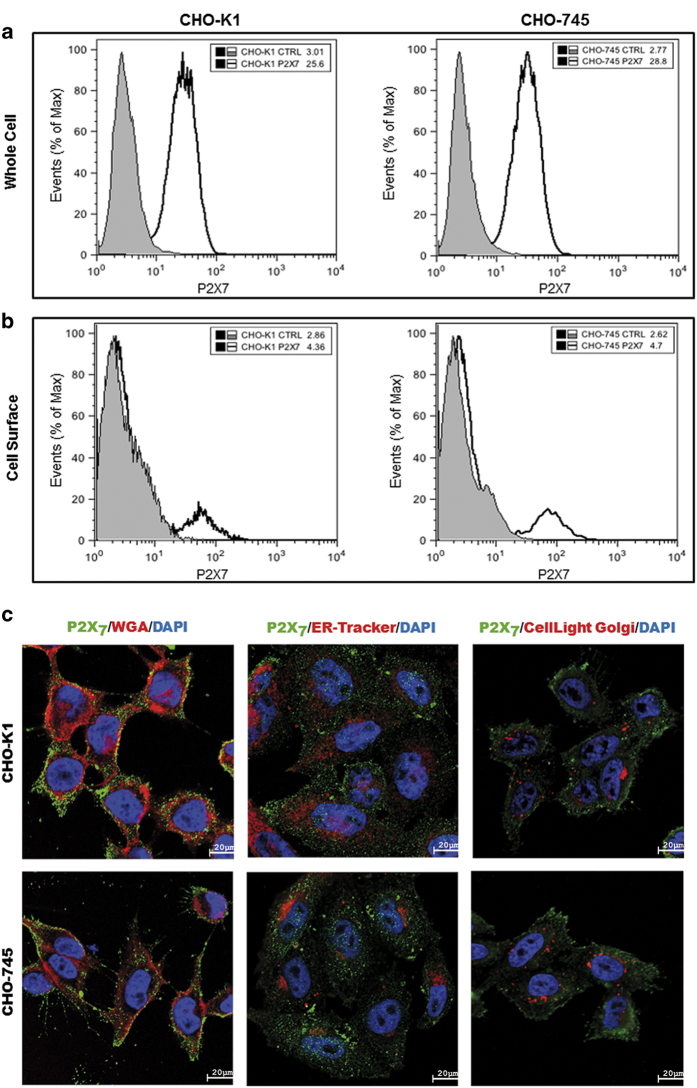
ATP-gated P2X_7_ receptor is expressed on the CHO cells surface. The P2X_7_ receptor expression was determined through flow cytometry analysis. CHO cells were labeled with antibody anti-P2X_7_ conjugated with Alexa Fluor 488, and the data were collected using a FACSCalibur flow cytometer (Becton–Dickinson) and analyzed using FlowJo software (Tree Star). The boundary between positive and negative cells labeled for the P2X_7_ receptor was determined according to the fluorescence distribution of positive cells relative to unlabeled control samples. (**a**) The amount of P2X_7_ receptor expressed in whole CHO cells. (**b**) P2X_7_ receptor expressed at the surface of CHO cells. (**c**) Immunofluorescence labeling of P2X_7_ in CHO-K1 and CHO-745 cells. Cells were stained with DAPI (blue) and immunolabelled with anti-P2X_7_ (green) and Alexa Fluor 594 conjugated to WGA (red) at left column; ER-Tracker (red) at central column; and with CellLight Golgi Fluorescent Protein (red) at right column. The histograms and images are representative of the results of three experiments. Scale bars, 20 *μ*m.

Alterations of the P2X_7_ response to the agonists ATP and BzATP observed in CHO-K1 and CHO-745 cells likely reflect either differences in P2X_7_ expression patterns or modulation mechanisms. Antibodies against the P2X_7_ receptor were used to investigate the cellular expression of this receptor using flow cytometry and confocal microscopy. The quantification of the P2X_7_ receptor expression through flow cytometry revealed that CHO-K1 and CHO-745 cells expressed similar amounts in the whole cell (permeabilized cells; Figure 3a). The quantification of only the P2X_7_ receptor external label (without permeabilization) also showed similar amounts in both cell lines (Figure 3b). These results suggest that the secretion of the P2X_7_ receptor was not altered in CHO-745 cells. To corroborate this hypothesis, confocal microscopy was performed after labeling the P2X_7_ receptor, cellular membrane, endoplasmic reticulum and Golgi (Figure 3c). We did not observe differences in P2X_7_ receptor pattern expression and cellular localization between CHO-K1 and CHO-745 cells (Figure 3c). These results indicate that the differences of Ca^2+^_cyt_ concentration curves in response to ATP and BzATP are not associated with the cellular expression or cellular localization of the receptor. Altogether, these data strongly suggest that the presence of GAGs on the surface of CHO cells modulates P2X_7_ receptor activity in response to agonists.

### The presence of GAGs at the CHO cell surface increases the efficacy and potency of P2X_7_ agonists

Concentration–response curves for ATP and the kinetics parameters, EC_50_, Hill coefficient and *E*_max_, were determined in wild-type CHO-K1 cells and mutant CHO-745 cells defective in GAG biosynthesis (Figure 1b). Interestingly, all the kinetic parameters of the ATP concentration–response curve were affected by the presence of GAGs on the CHO cell surface: potency (CHO-K1, EC_50_=3.2±0.3 *μ*M; CHO-745, 8±1 *μ*M), cooperativity (CHO-K1, Hill coefficient=0.9±0.1; CHO-745, 2.0±0.3) and maximal response (CHO-K1, *E*_max_=258±10 URF; CHO-745, 97±7 URF). The presence of GAGs significantly increased the sensitivity to low concentrations of ATP and altered the main P2X_7_ kinetic parameters (EC_50_, Hill coefficient and *E*_max_). Thus, the application of ATP to wild-type CHO-K1 cells increased the effectiveness of the P2X_7_ receptor and reduced the Hill coefficient from 2.0 to 0.9. Conversely, ATP when applied to CHO-745 cells, in the absence of GAG, decreased P2X_7_ effectiveness and increased the Hill coefficient from 0.9 to 2.0 (Table 1).

Similarly, GAGs also modulate the calcium influx evoked through BzATP in CHO cells (Figure 1d). The presence of GAGs enhanced the effectiveness of BzATP at all concentrations tested either by increasing the maximal BzATP response (CHO-K1, *E*_max_=275±8 URF; CHO-745, 136±6 URF) or by reducing the Hill coefficient from 1.8±0.2 to 1.0±0.1, although there were no changes in the EC_50_ (CHO-K1, 23±2 *μ*M; CHO-745, 21±3 *μ*M). Therefore, the application of BzATP to CHO-K1 cells increases P2X_7_ effectiveness, whereas for CHO-745 cells, the application of BzATP decreases P2X_7_ effectiveness and increases the Hill coefficient from 1.0 to 1.8 (Table 1).

As expected, Mg^2+^ changed the BzATP binding to the P2X_7_ receptor in an allosteric manner in CHO-K1 cells, where the BzATP maximal response was decreased (CHO-K1 control, *E*_max_=275±8 URF; 10 mM Mg^2+^, *E*_max_=153±6 URF), the Hill coefficient was increased (CHO-K1 control, 1.0±0.1; 10 mM Mg^2+^, 1.5±0,1) and the BzATP affinity for the receptor was strongly decreased (CHO-K1 control, 23±2 *μ*M; 10 mM Mg^2+^, 168±22 *μ*M). Interestingly, the application of 10 mM Mg^2+^ to CHO-745 cells did not change the P2X_7_ receptor maximal response (CHO-745 control, *E*_max_=136±6 URF; 10 mM Mg^2+^, *E*_max_=119±10 URF), but Mg^2+^ decreased P2X_7_ effectiveness (CHO-745 control, EC_50_=21±3 *μ*M; 10 mM Mg^2+^, EC_50_=150±11 *μ*M) and increased the Hill coefficient from 1.8±0.2 to 2.8±0.3. The presence of GAGs decreases the inhibitory effect of Mg^2+^ by increasing the maximal BzATP response and reducing the Hill coefficient, although no change in the EC_50_ values in the presence of Mg^2+^ was observed (Table 1).

### P2X_7_-mediated cell death is upregulated by GAGs

**Figure 4:**
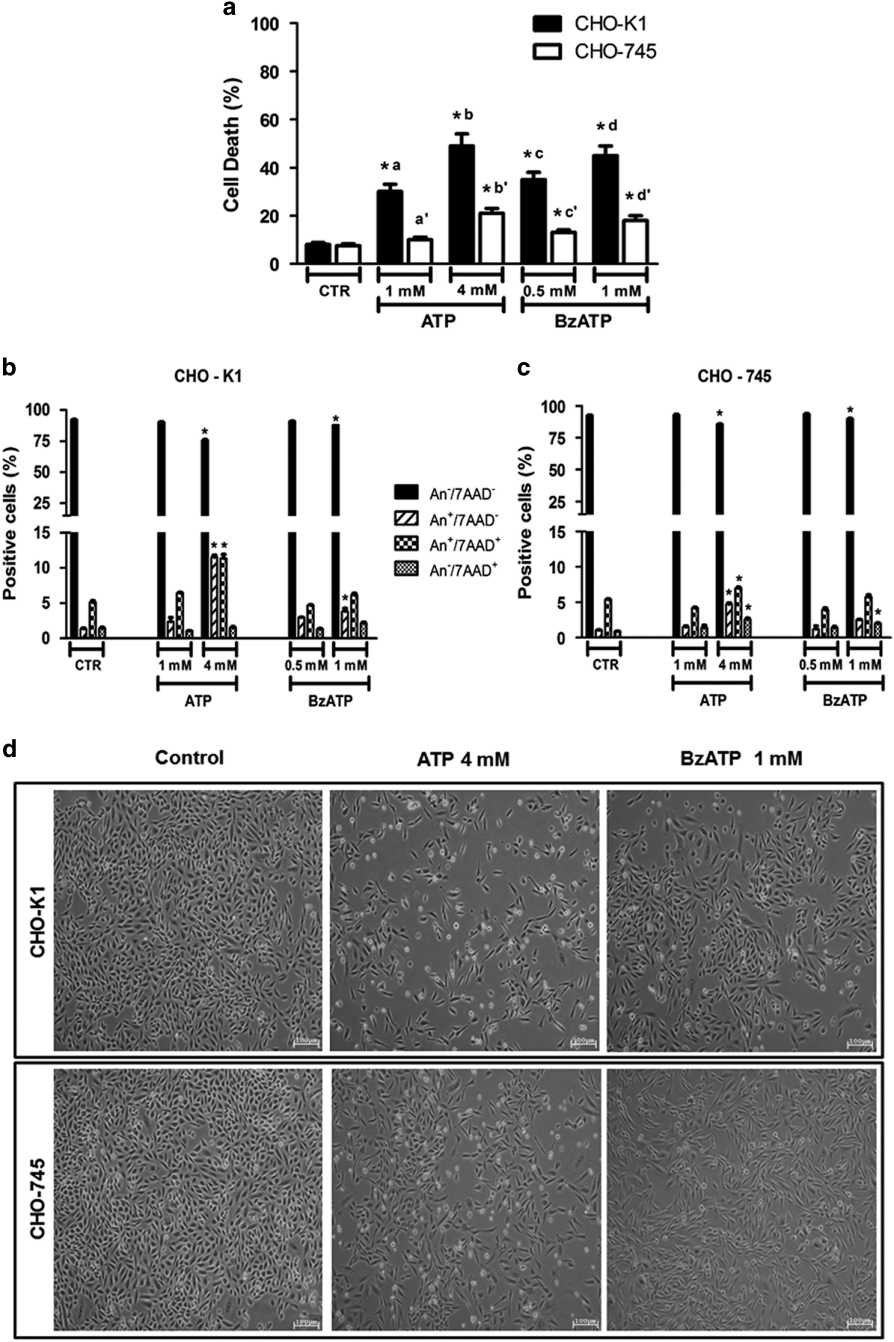
P2X_7_-mediated cell death is dependent of GAGs/Proteoglycans in CHO cells. (**a**) CHO-K1 and CHO-745 cells were incubated with ATP (1 and 4 mM) or BzATP (0.5 and 1 mM) for 24 h, at 37 ºC, and the viability of the CHO cell lines was determined using the MTT assay. (**b**, **c**) P2X_7_-mediated cell death in CHO-K1 and CHO-745 cells was also investigated using annexin V-APC/7-AAD double staining and analyzed through flow cytometry. The data represent the mean±S.E.M. (*N*=6), *P<0.05. (**d**) CHO cells were stimulated with 4 mM ATP or 1 mM BzATP for 24 h. ATP stimulation induces morphological changes and decreases the CHO cell number as observed in phase contrast microscopy.

The effect of ATP and BzATP on cell death was evaluated in both CHO cell lines; therefore, we compared the viability of CHO-K1 and CHO-745 cells after stimulation with high concentrations of P2X_7_ agonists for 24 h. The MTT (3-(4,5-dimethylthiazol-2-yl)-2,5-diphenyltetrazolium bromide) assay showed that a high concentration of ATP (>1 mM) and BzATP (>500 *μ*M) promoted cell death in both lines. However, differences in the percentage of cell death between CHO-K1 and CHO-745 cells were observed (Figure 4a). Similar differences in viability were also observed through flow cytometry using annexin V and 7-amino-actinomycin D (7-AAD; Figure 4b and c). These data showed that P2X_7_-mediated CHO cell death primarily occurs through the apoptosis pathway. Notably, wild-type CHO-K1 cells were 2.5-fold more sensitive to cell death induced through P2X_7_ agonists than the mutant CHO-745 cells defective in GAG biosynthesis. Moreover, ATP and BzATP increased morphological changes and cell detachment in CHO cultures (Figure 4d). Similar results were observed after 48 h of treatment with P2X_7_ agonists (Supplementary Figure SI2). These results reveal GAGs as positive regulators of P2X_7_ receptor pore formation, eventually leading to cell death.

### Modifications in GAG chains affect the response of P2X_7_ receptor

The present data suggest that the rate and extent of P2X_7_ GAG-induced sensitization determines the outcome of receptor activation. To corroborate this assumption, different approaches were used to examine the role of GAG chains upon P2X_7_ receptor function.

**Figure 5:**
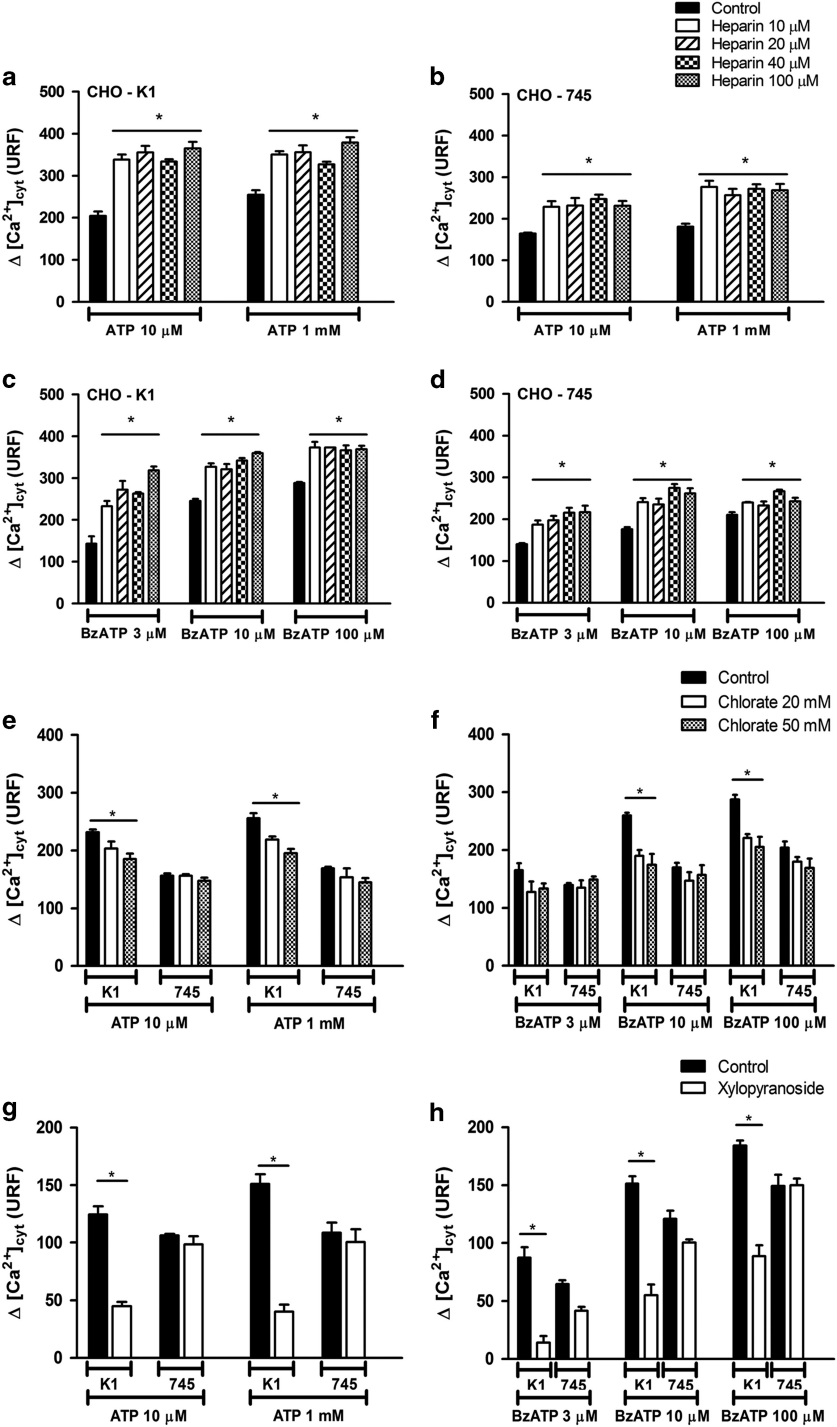
The enhancement in P2X_7_-mediated cytoplasmic Ca^2+^ influx is dependent on GAGs/proteoglycans in CHO cells. Concentration–effect curves for the ability of heparin (0–100 *μ*M) to modulate Ca^2+^ influx stimulated through ATP at concentrations of 10 *μ*M and 1 mM (**a** and **b**) or BzATP at concentrations of 3, 10 and 100 *μ*M (**c** and **d**) in CHO-K1 (**a** and **c**) and CHO-745 (**b** and **d**) cells. CHO-K1 and CHO-745 cells were pre-incubated with sodium chlorate, an inhibitor of PAPS, at concentrations of 20 and 50 mM for 48 h (**e** and **f**), or with 2 mM *o*-nitrophenyl-*β*-D-xylopyranoside, an inhibitor of endogenous proteoglycan biosynthesis, for 120 h (**g** and **h**); the CHO cells were subsequently stimulated with ATP (**e** and **g**) or BzATP (**f** and **h**). Cytoplasmic Ca^2+^ influx measurements in CHO cells were monitored through changes in the Fluo-4 fluorescence intensity in real time using the Flex Station 3 microplate reader system. The data represent the mean±S.E.M. (*N*=6). *P<0.05.

We used heparin, which raises the negative charge in the surrounding, mimicking endogenous GAGs chains on the receptor.^27,28^ We examined the effects of depleting or loading GAGs on P2X_7_ stimulation through the application of agonists. To examine the effects of heparin on the P2X_7_ response, the cells were pre-incubated with 10, 20, 40 or 100 *μ*M of heparin for 5 min before P2X_7_ agonist stimulation. Pre-treatment with heparin caused a larger increase in the amplitude of Ca^2+^ influx primarily evoked in lower concentrations of agonists (Figures 5a–d); for wild-type CHO-K1 cells, the increase was 120% Ca^2+^_cyt_ response at 10 *μ*M ATP and 78% at 1 mM ATP (Figure 5a). Similar results were obtained when the BzATP agonist was used. Notably, 100 *μ*M of heparin potentiated the Ca^2+^ influx induced through 3 *μ*M BzATP 2.2-fold (Figure 5c). However, the rate of sensitization through heparin was reduced in CHO-745 cells; for this group, the increase was only 40% of Ca^2+^ response at 10 *μ*M ATP and 50% at 1 mM ATP (Figure 5b). Similar rates of sensitization were obtained when CHO-745 cells were also stimulated with BzATP (Figure 5d). In the absence of ATP or BzATP, even the highest heparin concentration did not elicit P2X_7_ stimulation, and at saturation the agonist concentration (BzATP >100 *μ*M or ATP >4 mM) of heparin induced no further increase in Ca^2+^ influx. These results showed that heparin markedly sensitized Ca^2+^ entry through P2X_7_ at lower agonist concentrations (ATP and BzATP). These data suggest an allosteric sensitization mechanism of the receptor through heparin.

To deplete the GAG function in CHO cells, we treated these cells with 20 or 50 mM sodium chlorate for 24 h. We used chlorate to inhibit 3ʹ-phosphoadenosine-5ʹ-phosphosulfate synthase, which leads to the overall reduction in GAG sulfation.^29,30^ The pre-treatment of the wild-type CHO-K1 cells with chlorate inhibited ~35% of the total P2X_7_ agonist (ATP or BzATP) response. As expected, chlorate did not block the P2X_7_ agonist response in defective mutant CHO-745 cells (Figures 5e and f).

In addition, to selectively block GAGs biosynthesis, we treated CHO cells with o-nitrophenyl-*β*-D-xylopyranoside. Xylosides act as exogenous acceptors for the elongation of GAGs chains and thereby affect endogenous proteoglycans biosynthesis.^30,31^ The pre-treatment of CHO-K1 cells with 2 mM xylosides inhibited 55% of the response induced through 10 *μ*M ATP-gated P2X_7_ and 82% of the response induced through 3 *μ*M BzATP-gated P2X_7_ receptor. Moreover, as expected, xyloside did not block the P2X_7_ agonist response in mutant CHO-745 cells defective in GAG biosynthesis (Figures 5g and h). Altogether, these results showed that the perturbation of the GAG/proteoglycans biosynthesis greatly affects the P2X_7_ agonist response, revealing GAG/proteoglycans as an important physiological positive regulator of the native P2X_7_ receptor.

### Molecular association of P2X_7_ receptor and CD44 proteoglycans

**Figure 6:**
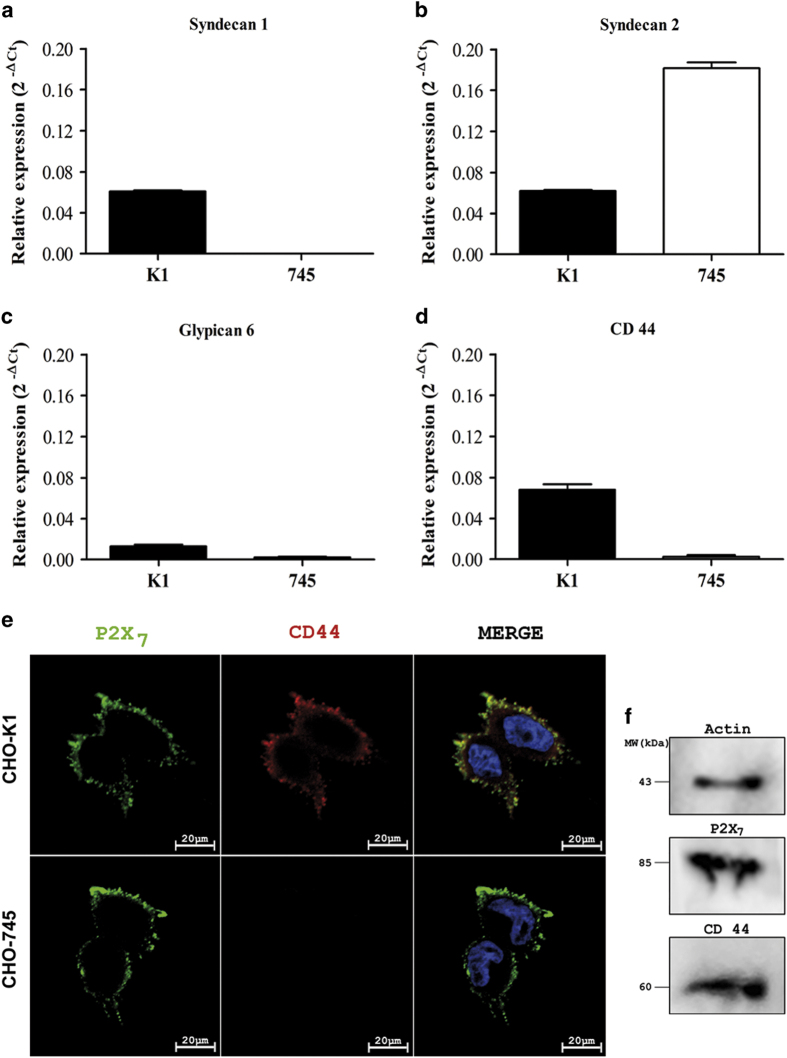
P2X_7_ and CD44 colocalize and closely interact in wild-type CHO-K1 cells. (**a**) Syndecan 1, (**b**) syndecan 2, (**c**) glypican 6 and (**d**) CD44 mRNA constitutive expression in CHO-K1 (black columns) and CHO-745 (white columns) cells, normalized over hypoxanthine–guanine phosphoribosyltransferase mRNA levels, and expressed as fold increases over controls (CTR, *n*=4). The data are represented as the means±S.E.M. (*N*=6). *P<0.05. (**e**) Immunofluorescence labeling with anti-P2X_7_ (green, first column) and anti-CD44 (red, second column) of CHO-K1 and CHO-745 cells. The nuclei were stained with DAPI (blue). Merge, third column. Scale bars, 20 *μ*m. (**f**) P2X_7_ immunoprecipitation of whole-CHO-K1 cell lysate. Aliquots (500 *μ*g) of cell lysates were incubated with polyclonal anti-P2X_7_ antibody overnight at 4 °C, followed by the addition of 20 *μ*l of protein A/G PLUS-Agarose for 4 h at 4 °C. The proteins were resolved through 10% SDS-PAGE and transferred onto polyvinylidene fluoride membranes. The immunoprecipitated proteins were detected using anti-P2X_7_ and anti-CD44 antibodies, followed by incubation with the respective secondary antibodies conjugated to horseradish peroxidase. The bands were revealed using the SuperSignal West Pico Chemiluminescent Substrate.

The CHO-K1 lineage expressed syndecan 1, syndecan 2, glypican 6 and CD44. The CHO-745 cells did not express syndecan 1, but expressed low levels of glypican 6 and CD44, and high levels of syndecan 2 (Figure 6a–d). In addition, both CHO cell lines did not express syndecan 3, syndecan 4, glypican 3, glypican 4 and glypican 5.

To evaluate the molecular association of P2X_7_ and CD44, confocal microscopy was performed. As expected, labeling revealed the co-localization of CD44 expression with the P2X_7_ receptor on the CHO-K1 cell surface (Figure 6d). This interaction was not observed in CHO-745 cells (Figure 6d). Furthermore, we used immunoprecipitation, followed by immunoblotting, to provide direct biochemical evidence for a physical association between the CD44 proteoglycan and the P2X_7_ receptor. Both, the P2X_7_ receptor and CD44, were individually identified in the complex molecular mass of 85 and 60 kDa, respectively (Figure 6e). The 60-kDa form of CD44 coimmunoprecipitated with the P2X_7_ receptor, corresponding with the soluble CD44 ectodomain (sCD44), a proteolytic product released from the membrane form of CD44 through shedding.^32^

### Heparin increases the dynamics of the P2X_7_ head domain

**Figure 7:**
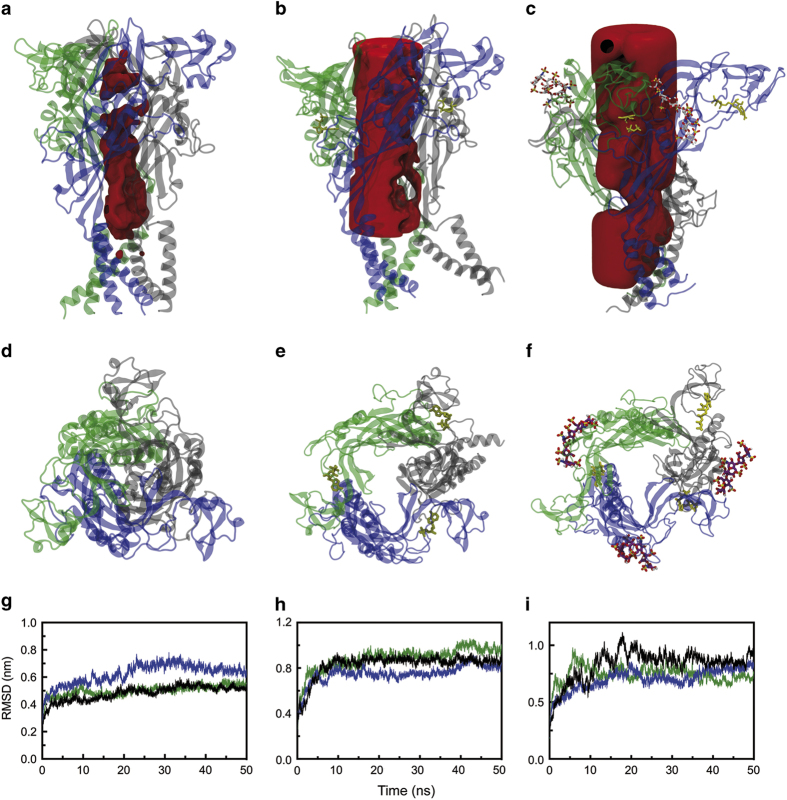
P2X_7_ molecular dynamics is altered through heparin binding. (**a**) P2X_7_ apo trimer, (**b**) P2X_7_ bound to ATP and (**c**) P2X_7_ bound to ATP and heparin (pink sticks). The overall shape of the P2X_7_ pore calculated as hollow under all conditions is shown in red. (**d**–**f**) Top view from **a**–**c**, respectively. (**g**–**i**) Ca RMSD for each individual subunit during the 50-ns dynamics simulations. Each ribbon color represents one chain from the representation above, ATP (yellow sticks) and heparin (pink sticks). The images were generated using VMD.

Here we proposed that the P2X_7_ channel dynamics are altered upon heparin binding. To examine this hypothesis, we investigated the relationship between the inherent dynamics of the head and tail after P2X_7_ apo, ATP-bound, heparin-bound and ATP–heparin complexes with the P2X_7_ receptor using docking, molecular dynamics and normal mode analysis (NMA). Figures 7a–f shows the channel volume after 50 ns calculation for the apo, P2X_7_-ATP and P2X_7_-ATP-heparin complexes as calculated through HOLLOW 1.2.^33^ Figures 7g–i shows the RMSD (root mean square deviation) fluctuations for each of the monomers of the P2X_7_ receptor during the 50-ns dynamics simulations. In the presence of ATP and ATP+heparin, an increase of ~0.2 and 0.4 Å, respectively, was observed in comparison with the apo structure, likely reflecting the increased relative motions after binding. To evaluate the influence of heparin binding in specific fluctuations of the P2X_7_ domains, we performed NMA. These simulations confirmed a more notable conformational fluctuation of the left flipper and tail domains in the heparin–ATP complex. These data strongly suggest that the processes to trigger pore dilation or the formation or recruitment of a pore are positively influenced through heparin, suggesting that heparin/GAGs, in the presence of ATP, promote the long opening-gating mode of the P2X_7_ receptor (Supplementary Figures SI3 and SI4).

## Discussion

Here we present evidence for a positive allosteric modulation of P2X_7_ through GAGs in CHO cells. These data strongly suggest that GAGs from the cell surface bind to the P2X_7_ receptor and thereby facilitates the binding of ATP to the ligand-gated cation channel. The presence of GAGs on the CHO cell surface greatly increases sensitivity to low concentrations of ATP and changes the main P2X_7_ kinetic parameters EC_50_, Hill coefficient and *E*_max_. In the absence of ATP, even the highest heparin concentrations tested did not elicit discernible P2X_7_ activation. The allosteric inhibition of the P2X_7_ receptor current through extracellular Mg^2+^ was mitigated in the presence of GAGs. These data suggest the allosteric sensitization of the receptor through GAGs. In addition, the formation, recruitment and dilation of the P2X_7_ pore augmented in the presence of GAGs as demonstrated through the acceleration of cellular uptake of the large molecule PI (MW 668) and molecular dynamic simulations. Increases in *E*_max_ of [Ca^2+^]_cyt_ and acceleration of PI influx confirmed the potentiating effect of GAGs on native P2X_7_ receptors. Consequently, wild-type CHO-K1 cells were 2.5-fold more sensitive to cell death induced through P2X_7_ agonists compared with mutant CHO-745 cells defective in GAG biosynthesis.

The ability of P2X_7_ to respond to a large range of ATP concentrations reflects ATP binding to the three sites on the trimeric receptor with negative cooperativity.^34^ Where partial ATP occupancy results in the opening of an intrinsic nonselective pore for small mono- and divalent cations, including Ca^2+^, full occupancy at high ATP concentrations triggers the dilation of the pore. Thus, the rate and extent of P2X_7_ sensitization determines the outcome of receptor activation. In the present study, we identified cell surface GAGs as key regulators of P2X_7_ receptor sensitization and pore dilation. These data support a model that GAG binding might overcome the conformational hindrances under conditions of partial agonist occupancy and thereby promote the long opening-gating mode.

Recently, the modulation of the P2X_7_ receptor through hyaluronic acid has also been observed in wound healing^35^ and ophthalmic cells.^36^ Moreover, soluble biglycan proteoglycan, released through proteolysis from the extracellular matrix, acts as a fundamental danger signal through interactions with Toll-like and purinergic P2X_4_/P2X_7_ receptors on the cell surface of macrophages.^9^ Together, these data showed that GAG chains from proteoglycans are new physiologically relevant binding partners from the cell surface microenvironment that activate the P2X_7_ receptor through an allosteric upregulatory mechanism. Consistent with these data, we demonstrated that P2X_7_ receptors interact with CD44 heparan sulfate proteoglycan on the wild-type CHO-K1 cell surface but not in the CHO-745 line.

CD44 proteoglycan is the major receptor of the extracellular matrix component hyaluronan,^37^ which acts as a co-receptor for growth factors and organizes the actin cytoskeleton through cell signaling. CD44 is involved in a wide spectrum of physiological functions, such as cell–cell and cell–matrix interactions, morphogenesis, cell migration, cellular differentiation and tumorigenic process.^38^ CD44 proteoglycan^39^ and P2X_7_ receptor^40^ are located in lipid raft regions at cell membrane. Low cholesterol level in lipid raft triggers membrane-dependent CD44 shedding^41^ and induces P2X_7_ receptor activation,^42^ suggesting that these biological processes might be associated. Moreover, extracellular ATP induces CD44 shedding from the cell surface via the activation of the P2X_7_ receptor, showing a functional relationship between CD44 and P2X_7_ receptors in macrophage-like P388D1 cells.^43^

Tumorigenic cells overexpressing P2X_7_ receptor show an increased tendency to metastasize,^44,45^ and paralleling CD44 expression is essential for the anchorage-independent growth and tumor-initiating ability of highly tumorigenic mammary epithelial.^46^ Notably, CD44 proteolytic cleavage serves as a tumorigenic process by enhancing the proliferation/migration of cells.^47^ The extracellular sCD44 proteolytically released domain affects the function of the membrane form of CD44, acting as an inhibitor of CD44-dependent cell–cell and cell–matrix interactions.^48,49^

Interestingly, ATP-mediated cytoplasmic Ca^2+^ influx through P2X_7_ receptors induces calpain activation.^50–53^ Activated calpain cleaves merlin, a Ezrin/Radixin/Moesin (ERM)-like protein.^54^ Recently, Hartmann^47^ showed that merlin activation through dephosphorylation prevents the proteolytic cleavage of CD44. In addition, P2X_7_ receptor stimulation also triggers *α*-secretase-dependent proteolytic shedding^55^ through phosphorylation of ERM proteins.^56^ Altogether, these data suggest that P2X_7_ receptor stimulation promotes CD44 shedding either through the direct activation of calpain or through the indirect phosphorylation of ERM proteins. Notably, calpain activation results in merlin cleavage, paralleling ERM phosphorylation, which promotes the activation of *α*-secretase protease, and both processes result in CD44 shedding.

Here, we provided the first evidence that the P2X_7_ receptor is positively modulated through GAG and interacts with CD44 on the CHO-K1 cell surface. Moreover, we also showed that the P2X_7_ receptor coimmunoprecipitates with the sCD44 ectodomain, sCD44, in CHO-K1 lysates. We did not observe this interaction on the surface of CHO-745 cells, indicating that GAG chains mediate the interaction of sCD44 proteoglycans with the P2X_7_ receptor. Therefore, the present study proposes sCD44 as a new physiologically positive allosteric modulator of the P2X_7_ receptor; sCD44 is part of a regulatory positive feedback loop linking P2X_7_ receptor activation and thereby facilitating the intracellular response mediated through ATP cell signaling.

## Materials and Methods

### Materials

Nutrient mixture F12 medium, fetal bovine serum (FBS), penicillin, streptomycin and trypsin, the agonists ATP, ADP, BzATP, UDP, UTP, 2-MeSATP, *αβ*-MeATP and *βγ*-MeATP, the antagonist oxATP and other chemicals, such as dimethyl sulfoxide (DMSO) and MTT, were purchased from Sigma-Aldrich (St. Louis, MO, USA). Annexin V-APC (An) and 7-AAD were obtained from BD Biosciences (San Jose, CA, USA). Fluo-4 NW Calcium Assay kit was obtained from Molecular Probes (Eugene, OR, USA). Protein A/G PLUS-Agarose and anti-P2X_7_ receptor and anti-CD44 antibodies were purchased from Santa Cruz Biotechnology (Dallas, TX, USA). IgG-conjugated antibodies with horseradish peroxidase were obtained from Cell Signaling Technology (Danvers, MA, USA). SuperSignal West Pico Chemiluminescent Substrate and micro BCA were purchased from Thermo Scientific (Waltham, MA, USA). TRIzol reagent, 4ʹ,6-diamidino-2-phenylindole (DAPI), secondary antibodies conjugated with Alexa Fluor and fluorescents organelles markers, such as ER-tracker red, CellLight Golgi red and Alexa Fluor 594-conjugated wheat germ agglutinin (WGA), were obtained from Invitrogen (Waltham, MA, USA). The ImProm-II Reverse Transcriptase System kit was purchased from Promega (Madison, WI, USA), SYBR Green PCR Master Mix was obtained from Applied Biosystems (Carlsbad, CA, USA) and the primers were obtained from Integrated DNA Technologies (Coralville, IA, USA).

### Cell lines and culture conditions

This research was conducted using established cell lines. Wild-type CHO-K1 cells and mutant CHO-745 cells, deficient in xylosyltransferase, resulting in deficient proteoglycan biosynthesis, were kindly donated from Professor Dr Jeffrey D Esko (Glycobiology Research and Training Center, University of California, San Diego La Jolla, CA, USA). The CHO cells were cultured in F12 medium containing 10% (v/v) heat-inactivated FBS, 10 U/ml penicillin and 10 *μ*g/ml streptomycin at 37 °C in an atmosphere of 5% (v/v) CO_2_.

### Measurement of cytoplasmic Ca^2+^

For cytoplasmic Ca^2+^ concentration measurements, CHO cells were seeded onto black 96-well plates (10^4^ cells/well) and maintained for 48 h at 37 °C under an atmosphere of 5% (v/v) CO_2_. Subsequently, the cells were incubated with Fluo-4 Direct Calcium Assay reagent for 1 h at 37 °C, according to the manufacturer’s instructions. The samples were stimulated with different agonists, and the fluorescence was quantified using a Flex Station 3 microplate reader (Molecular Devices, Sunnyvale, CA, USA). The Fluo-4 was excited at 490 nm and the emission was detected at 525 nm.^57^

### Flow cytometry analysis

P2X_7_ receptor expression was determined using flow cytometry analysis. CHO cells were seeded onto six-well plates and maintained for 48 h at 37 °C under an atmosphere of 5% (v/v) CO_2_. Subsequently, the cells were harvested using 10 mM EDTA in phosphate-buffered saline (PBS) and adjusted to a concentration of 10^6^ cells per tube. Initially, to examine the protein expression at the cell surface, the cells were incubated with goat anti-P2X_7_ primary antibodies, washed with PBS buffer and labeled with the respective secondary antibody conjugated to Alexa Fluor 488. In addition, to label the P2X_7_ receptor on whole cells, before the procedure mentioned above, the cells were fixed in 2% paraformaldehyde/PBS for 30 min, washed three times with 0.1 M glycine/PBS and permeabilized with 0.01% saponin/PBS for 15 min. The data were collected using a FACSCalibur flow cytometer (Becton–Dickinson, Franklin Lakes, NJ, USA) and CellQuest software (Becton–Dickinson), followed by analysis using FlowJo software (Tree Star, CA, USA). A total of 10 000 events were collected for each sample. The boundary between the cells positive and negative for P2X_7_ receptor labeling was determined according to the fluorescence distribution of the positive cells relative to the control (unlabeled samples).

### Cell viability assay

The viability of CHO cell lines was determined using the MTT assay.^58^ Briefly, the cells were seeded onto 96-well plates (10^4^ cells/well) and maintained for 48 h at 37 °C under an atmosphere of 5% (v/v) CO_2_. Subsequently, the medium was removed and the cells were incubated with ATP (1 or 4 mM) or BzATP (0.5 or 1 mM) at a final volume of 200 *μ*l/well in fresh medium for 24 and 48 h. The medium was removed, 100 *μ*l of MTT (1 mg/ml) was added to each well and the cells were incubated again at 37 °C for 4 h. The supernatant was removed and 200 *μ*l/well of DMSO was added to solubilize the formazan crystals formed. The absorbance was read at 570 nm using a microplate reader, and the cell viability was determined using the standard reduction of the MTT. The results were expressed relative to control cell viability (100%).

### Cell death assay

Cell death was investigated using annexin V-APC/7-AAD double staining and was analyzed through flow cytometry.^57,59^ The cells were seeded onto six-well plates (10^6^ cells/well) and maintained for 48 h at 37 °C under an atmosphere of 5% (v/v) CO_2_. Subsequently, the medium was removed, and the cells were incubated with ATP (1 or 4 mM) or BzATP (0.5 or 1 mM) for 24 and 48 h, followed by harvesting with trypsin, washing with PBS and resuspending in binding buffer (0.01 M HEPES, pH 7.4, 140 mM NaCl and 2.5 mM CaCl_2_). The suspensions were transferred to tubes, centrifuged and resuspended with annexin V-APC and 7-AAD according to the manufacturer’s instructions. The cells were incubated at room temperature for 30 min, and the viable, apoptotic or necrotic cell populations were evaluated through flow cytometry. The data were collected using a FACSCalibur flow cytometer (Becton–Dickinson) and CellQuest software (Becton–Dickinson), followed by analysis using FlowJo software (Tree Star). A total of 10 000 events were collected for each sample. The results were calculated as the percentage of cell death compared with the control.

### Dye uptake assay

P2X_7_ pore formation was functionality examined in CHO cells by analyzing PI uptake using a FACSCalibur flow cytometer (Becton–Dickinson). The cells were seeded onto six-well plates (10^6^ cells/well) and maintained for 48 h at 37 °C under an atmosphere of 5% (v/v) CO_2_. Subsequently, the cells were harvested using 10 mM EDTA/PBS and adjusted to a concentration of 10^6^ cells per tube. The samples were incubated with 5 *μ*M of PI at a final volume of 200 *μ*l/well in fresh serum-free medium F12 for 15 min, followed by the addition of 4 mM ATP and incubation at 37 °C for 30, 60 and 120 min, respectively. The intensity of dye uptake was immediately determined. A total of 10 000 events were collected for each sample.^60,61^

### Quantification of gene expression

Total RNAs were extracted using TRIzol reagent, and the reverse-transcribed cDNA was obtained using the ImProm-II Reverse Transcriptase System kit according to the manufacturer's protocol. Real-time quantitative PCR assays were performed using the SYBR Green PCR Master Mix, and the primers are described in Supplementary Table SI1. The cycling parameters for the PCRs were 50 °C for 2 min, 95 °C for 10 min, followed by 40 cycles of 95 °C for 15 s and 60 °C for 1 min in an ABI PRISM 7500 Real Time PCR System (Applied Biosystems). Standard curves were obtained for each primer pair to assess the efficiency of amplification. The target mRNA expression was normalized to the housekeeping gene enzyme hypoxanthine–guanine phosphoribosyltransferase, and the relative quantification of the expression levels (experimental/control) was determined based on the 2-[Δ]Ct method.^62^

### Confocal immunofluorescence assay

For analysis of expression pattern and cellular localization of the P2X_7_ receptor through confocal microscopy, CHO cells were seeded (10^4^ cells) onto coverslips until reaching ~60% confluence. Initially, to examine protein expression at the cell surface, the cells were washed three times with PBS, incubated with goat anti-P2X_7_ primary antibody and subsequently developed using the respective secondary antibody conjugated to Alexa Fluor 488. Thereafter, the cells were washed three times with PBS, and labeled with rat anti-CD44 primary antibody, followed by incubation with the respective secondary antibody conjugated to Alexa Fluor 594 or the cell membrane-specific marker, WGA, conjugated to Alexa Fluor 594. The cells were fixed with 2% paraformaldehyde/PBS for 30 min, washed three times with 0.1 M glycine/PBS, permeabilized with 0.01% saponin/PBS for 15 min and stained with DAPI for 15 min. The coverslips were mounted onto microscopy slides using Fluoromont G (Immunkemi, Stockholm, Sweden). In addition, to analyze the P2X_7_ receptor localization in intracellular compartments, the CHO cells were labeled with ER-Tracker red, a endoplasmic reticulum-specific marker, or transfected with CellLight Golgi red fluorescent protein, which is exclusively expressed in the Golgi, according to the manufacturer’s instructions. Subsequently, the cells were washed, fixed, permeabilized and labeled with the anti-P2X_7_ receptor antibody, as described above. Light microscopy analysis was performed using a confocal laser-scanning Zeiss LSM 780 microscope (Zeiss, Oberkochen, Germany). The pinhole device was adjusted to capture the fluorescence of one airy unit. The images were processed using ImageJ software (National Institutes of Health, Bethesda, MD, USA).

### Immunoprecipitation assay and western blot analysis

The cells were lysed with the non-denaturing lysis buffer containing 20 mM Tris, pH 7.4, 2 mM EDTA, 137 mM NaCl, 1% Nonidet-P40 and protease/phosphatase inhibitor cocktail. Aliquots (500 *μ*g) of the lysates were incubated with polyclonal anti-P2X_7_ antibody overnight at 4 °C, followed by the addition of 20 *μ*l of Protein A/G PLUS-Agarose reagent for 4 h at 4 °C. Immune complexes were collected through centrifugation and washed three times with PBS. After the final wash, the supernatant was discarded, and the pellet was dissolved in SDS lysis buffer and boiled in fivefold SDS loading dye for 5 min. The proteins were resolved through 10% SDS-PAGE and transferred to polyvinylidene fluoride membranes. Immunoprecipitated proteins were subsequently detected using anti-P2X_7_ and anti-CD44 antibodies, followed by incubation with the respective secondary antibodies conjugated to horseradish peroxidase. The bands were revealed using the SuperSignal West Pico Chemiluminescent Substrate.^63^

### Molecular dynamics and free-energy calculation

#### Model building

The structure of the P2X_7_ receptor was constructed through homology modeling using the software program Modeler6v2,^64^ using the P2X_4_ receptor as template, Protein Data Bank entry code 3H9V. The model was energy-minimized (100 cycles of steepest descent) using GROMACS-4.5.1,^65,66^ and submitted to 50 ns dynamics simulation.

#### Heparin and ATP topology construction

The heparin coordinates and topologies were generated as previously described.^67,68^ Briefly, saccharides composing fragments were constructed using MOLDEN software,^69^ and the structures were submitted to the PRODRG server.^70^ Thereafter, using minimized output conformations, a series of MD simulations were performed for 20 ps at 10 K, with an integration step of 0.5 fs, to further reinforce the search for minimum-energy states. Relaxed structures were used to perform docking experiments. ATP topology is part of the GROMOS96 43a1 force field.^71^

#### Docking

The location of ATP was determined through superimposition with 3H9V using the software program Modeler6v2. The docking of heparin onto the P2X_7_ receptor models (P2X_7_ alone and P2X_7_ containing ATP) was performed using the software program Autodock 4.2.^72^ The Löwdin atomic charges for sulfated saccharides were used,^73,74^ and all torsion angles were considered to be flexible. The grid maps were calculated using AutoGrid, and the grid dimensions were set to 160 Å×150 Å×80 Å, with 0.3 Å spacing between the grid points. The region between two monomers was also considered during the docking runs. For each simulation, 500 runs of genetic algorithm were performed using a population of 1000 individuals, a maximum number of 2.5×10^8^ energy evaluations, a 0.02 mutation rate, a 0.80 crossover rate and an elitism value of 1. The results were clustered according to the 0.4-Å RMSD criteria and the Autodock score.

#### Molecular dynamics simulations

Molecular dynamics simulation was performed using GROMACS-4.5.1 with the GROMOS96 43a1 force field. The P2X_7_ trimer alone, ATP-bound or ATP/heparin-bound was solvated in rectangular boxes using periodic boundary conditions and the SPC water model.^75^ Counter ions (Na^+^ and Cl^−^) were added to neutralize the system, whenever needed. The employed MD protocol was based on previous studies.^67^ The Lincs method^76^ was applied to constrain covalent bond lengths, allowing an integration step of 2 fs after an initial energy minimization using the Steepest Descents algorithm. Electrostatic interactions were calculated using the Particle Mesh Ewald method.^77^ Temperature and pressure were maintained through the coupling protein, heparin, ATP, ions and solvent to external temperature and pressure baths with coupling constants of *τ*=0.1 and 0.5 ps, respectively. The dielectric constant was treated as *ε*=1. The systems were slowly heated from 50 to 310 K, in steps of 5 ps, each increasing the reference temperature by 50 K. After heating, all simulations were further extended to 50 ns under a constant temperature of 310 K. The hydrogen bonds were defined when the donor–acceptor heavy atom distance was 0.35 nm and the acceptor atom–donor hydrogen angle was 30 degrees. Molecular dynamics simulations were performed at the Ohio Supercomputer Center Oakley Cluster, using a 16-processors system of 49 052 atoms at a rate of ~2.1 *μ*s/atom/timestep. Trajectories were acquired every 10 ps and were visualized using VMD v1.9.1^78^ and Pymol.^79^

#### MM-PBSA binding free-energy calculation

Binding free-energy (DGbind) calculations were performed using g_mmpbsa package.^80^ The trajectories were compressed every 100th frame, and all three heparin-bound monomers were analyzed. All 2D data were plotted using Grace 5.1 software (Free Software Foundation, Boston, MA, USA).

### Statistical analysis

All data for each assay represent two independent experiments run at least in triplicate (*N*=6), and are expressed as the mean±S.E.M. Statistical significance among the groups was assessed using one-way analysis of variance ANOVA, followed by Tukey’s multiple comparison test. Student’s *t*-test was also used to analyze the data when pertinent. Differences were considered to be significant when the *P-*values were <0.05 (*P*<0.05). All statistical analyses were performed using GraphPad Prism software (GraphPad, La Jolla, CA, USA).

## Figures and Tables

**Table 1:** Kinetic parameters of P2X_7_ receptor in CHO cells

*Agonist*	*CHO-K1*			*CHO-745*		
	*E_max_ (URF)*	*EC_50_* *(*μ*M)*	*Hill coefficient*	*E*_*max*_ *(URF)*	*EC*_*50*_ *(*μ*M)*	*Hill coefficient*
ATP	258±10	3.2±0.3	0.9±0.1	97±7	8±1	2.0±0.3
BzATP	275±8	23±2	1.0±0.1	136±6	21±3	1.8±0.2
BzATP+Mg^2+^	153±6	168±22	1.5±0.1	119±10	150±11	2.8±0.3

## Abbreviations

*α**β*-MeATP: *α*,*β*-methyleneadenosine 5′-triphosphate
*β**γ*-MeATP: *β*,*γ*-methyleneadenosine 5′-triphosphate
2-MeSATP: 2-(methylthio)adenosine 5′-triphosphate
7-AAD: 7-amino-actinomycin D
BzATP: 2′(3′)-O-(4-benzoylbenzoyl)adenosine 5′-triphosphate
CHO: Cell line derived from the ovary of the Chinese hamster
DAPI: 4ʹ-6-diamidino-2-phenylindole
DMSO: dimethyl sulfoxide
ERM: Ezrin/Radixin/Moesin family
FBS: fetal bovine serum
GAG: glycosaminoglycans
MTT: 3-(4,5-dimethylthiazol-2-yl)-2,5-diphenyltetrazolium bromide
NMA: normal mode analysis
OxATP: adenosine 5′-triphosphate periodate oxidized
P2X: ATP-gated cation channel receptors family
P2X_7_: purinergic receptor P2X, ligand-gated ion channel 7
P2Y: G-protein-coupled receptors family
P388D1: macrophage-like cell line derived from murine
PI: propidium iodide
RMSD: root mean square deviation
sCD44: soluble CD44 ectodomain
TGF-*β*: transforming growth factor-beta.
