# Supplementary material for: Post-translational allosteric activation of the P2X7 receptor through glycosaminoglycan chains of CD44 proteoglycans
Source: Cell Death Discov. 2015 Oct 5;1:15005–. doi: 10.1038/cddiscovery.2015.5 (PMC4979527; doi:10.1038/cddiscovery.2015.5)
Supplement: Supplementary Information [file cddiscovery20155-s1.doc]

**SUPPLEMENTARY INFORMATION**

1. **SUPPLEMENTARY RESULTS: MOLECULAR DYNAMICS OF THE P2X_7_ RECEPTOR**

Figure SI3A-C shows the residue root mean square fluctuations in the conditions studied, with relative NMA frequencies (Fig SI3D-F). Upon heparin binding, a clear increase in the motions of the head domain (A-C, number 1, yellow arrows) and rotation around the central axis of protein (D-F, black arrows) are observed. After ATP binding, there is a notable increase in conformational fluctuations of the head domain compared with other regions of the protein. In addition to the motion of the head domain, the region along the left flipper shows a higher degree of motion (Fig SI4A – pink) compared with both apo (black) and ATP-bound complexes (Fig SI4A – orange), suggesting that heparin increased the inherent conformational fluctuations of the head domain, and together with experimental data, might explain the allosteric changes experimentally observed *in vitro*. As previously demonstrated for the P2X4.1 receptor , the transition from closed to open pore trigged through ATP and heparin binding greatly alters intra- and intersubunit interactions between the TM2 helices in the region of pore constriction of the P2X_7_ receptor. Val335 and Ser342 represent the extracellular and intracellular ends of the gate that restricts ion flow in the closed state of the TM2 helices in the ion-conducting pathway, corresponding to Leu340 and Ala347 in the zebrafish P2X4.1R . The molecular dynamic of the intersubunit distance between the Cα position of Val335 and Cα position of Ser342 are shown (Fig SI4B) for apo P2X_7_ (line black), the P2X_7_-ATP complex (line blue) and the P2X_7_-ATP-heparin complex (green). In the closed state, after 50 ns of simulation, the intersubunit distance Val335-Ser342 stabilizes approximately 0.8 ± 0.2 nm. ATP binding promotes the time-dependent modulation of the intersubunit distance Val335-Ser342, first causing a rapid increase of this distance (2.5 ± 0.5 nm), followed by a new open state after 13 ns of simulation (5 ± 1 nm), suggesting the intrinsic transient dilation of the P2X_7_ pore (line Blue). These data support the current hypothesis that the occupancy of the ATP binding sites of P2X_7_ receptor first cause the rapid opening of a small cation-permeable pore, followed by a second gradual dilation that renders the pore permeable to large organic dyes . The presence of heparin in P2X_7_-ATP complex promotes the rapid expansion of the intersubunit distance Val335-Ser342 (6.0 ± 1.5 nm), showing facilitation in the dilation of the P2X_7_ pore (line green). We used MM-PBSA after processing 100 collected snapshots for each of the dynamics simulations, and the relative binding free energy DG*binding can be calculated for assessing the quality and validity of the resulting P2X_7_-ATP-heparin complexes (Fig SI4C). Relative energies for each heparin-monomer were calculated for P2X_7_-heparin (dashed lines) and P2X_7_-ATP-heparin (solid lines) complexes. As expected, the presence of ATP positively modulates the binding of heparin on P2X_7_ receptor. Altogether, DM simulation data showed that heparin induced/enforced downstream motions of the head domain reflect opening-related allosteric changes, such as the radial expansions of extracellular vestibule and the final iris-like channel opening.

1. **SUPPLEMENTARY INFORMATION: REFERENCES**

1. Hattori M, Gouaux E. Molecular mechanism of ATP binding and ion channel activation in P2X receptors. *Nature*. 2012; **485**: 207-12.

2. Jiang R, Taly A, Grutter T. Moving through the gate in ATP-activated P2X receptors. *Trends in biochemical sciences*. 2013; **38**: 20-9.

3. Rokic MB, Stojilkovic SS. Two open states of P2X receptor channels. *Front Cell Neurosci*. 2013; **7**: 215.

1. **SUPPLEMENTARY INFORMATION: FIGURE LEGENDS**

**FIGURE SI1:** **Time course of cytoplasmic Ca^2+^ influx elicited by ATP or BzATP in CHO cells.** Time course curves of cytoplasmic Ca^2+^ influx in CHO-K1 (*A, C*) and CHO-745 (*B*, *D*) cells in response to different concentrations of ATP-gated P2X_7_ (*A*, *B*) or BzATP-gated P2X_7_ (C, D). Time course of cytoplasmic Ca^2+^ influx in CHO cells were monitored through changes of the Fluo-4 fluorescence intensity in real time using the FlexStation 3 microplate reader system as previously described in the Materials and methods section.

**FIGURE SI2: P2X_7_-mediated cell death is dependent of GAGs/Proteoglycans in CHO cells.** *A*) CHO-K1 and CHO-745 cells were incubated with ATP (1 mM; 4 mM) or BzATP (0.5 mM; 1 mM) for 48 h at 37ºC under an atmosphere of 5% (v/v) CO_2_, and the viability of CHO cell lines was determined using MTT assays; the data represent the means ± SEM (N = 6), *P < 0.05.  *B*) P2X_7_-mediated cell death in CHO-K1 and CHO-745 cells was also investigated using annexin V-APC/7-AAD double staining and analysed through FACS. The data represent the means ± SEM (N = 6), *P < 0.05. *C*) P2X_7_ receptor stimulation with 4 mM ATP or 1 mM BzATP for 48 h induces morphological changes and decreases the CHO cell number as observed under phase contrast microscopy.

**FIGURE SI3. Heparin binding affects P2X_7_ receptor motions.** General motions detected through NMA and MD simulations. *A-C*) R.M.S.F fluctuations of various domains during MD simulations. Each colour represents one P2X_7_ receptor monomer. The numbers indicate NMA modes (arrow vectors). *D-F*) Vectors representing the amplitude and direction of the displacements experienced in residues during the conformation changes.

**FIGURE SI4. Averaged R.M.S.** *A*) Fluctuations of P2X_7_ apo (black) P2X_7_/ATP complex (orange) and P2X_7_/ATP/HEPARIN (pink). *B*) Averaged Leu-ALa distances during the 50 ns dynamics simulations of P2X_7_ apo (Black), P2X_7_/HEP (Blue), and P2X_7_/ATP/HEP (Green). *C*) Heparin binding energies as calculated using MM-PBSA. Black, blue and green lines represent energies for each monomer in the presence of ATP. Pink, red and brown lines represent energies for each monomer in the absence of ATP.
