# Supplementary figures and images for: Post-translational allosteric activation of the P2X7 receptor through glycosaminoglycan chains of CD44 proteoglycans
Source: Cell Death Discov. 2015 Oct 5;1:15005–. doi: 10.1038/cddiscovery.2015.5 (PMC4979527; doi:10.1038/cddiscovery.2015.5)

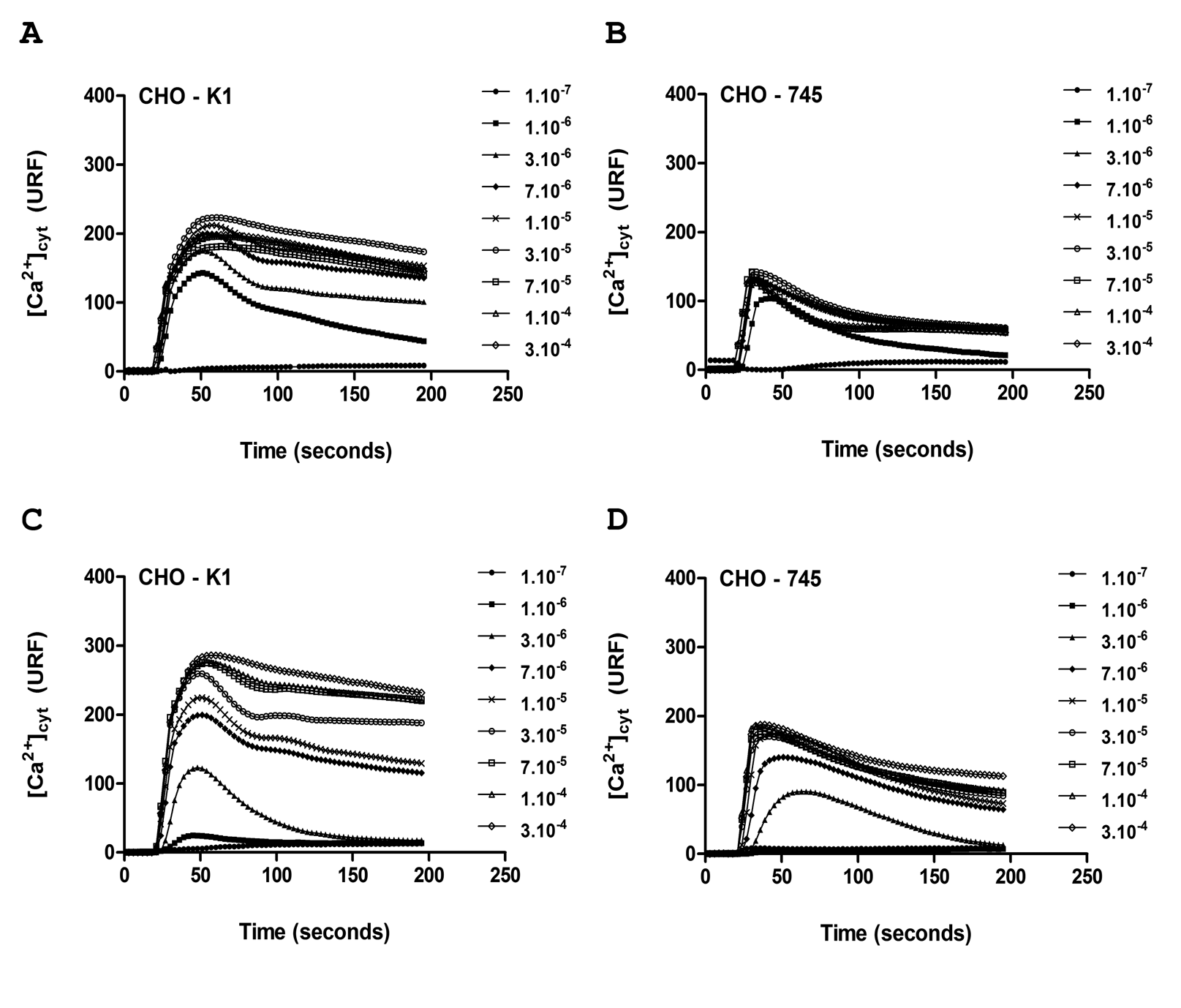

Supplement: Supplementary Figure 1 [file cddiscovery20155-s2.tiff]

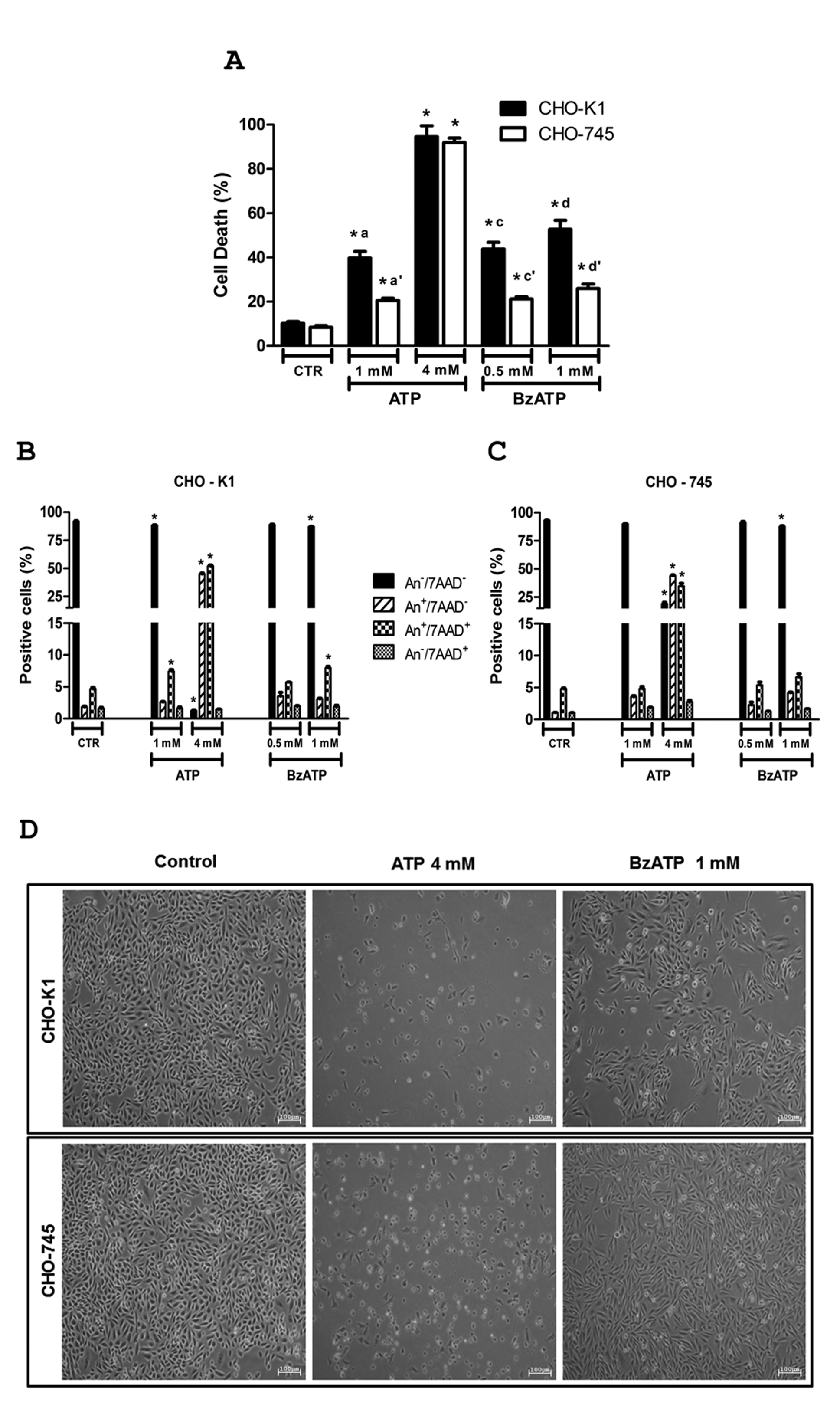

Supplement: Supplementary Figure 2 [file cddiscovery20155-s3.tiff]

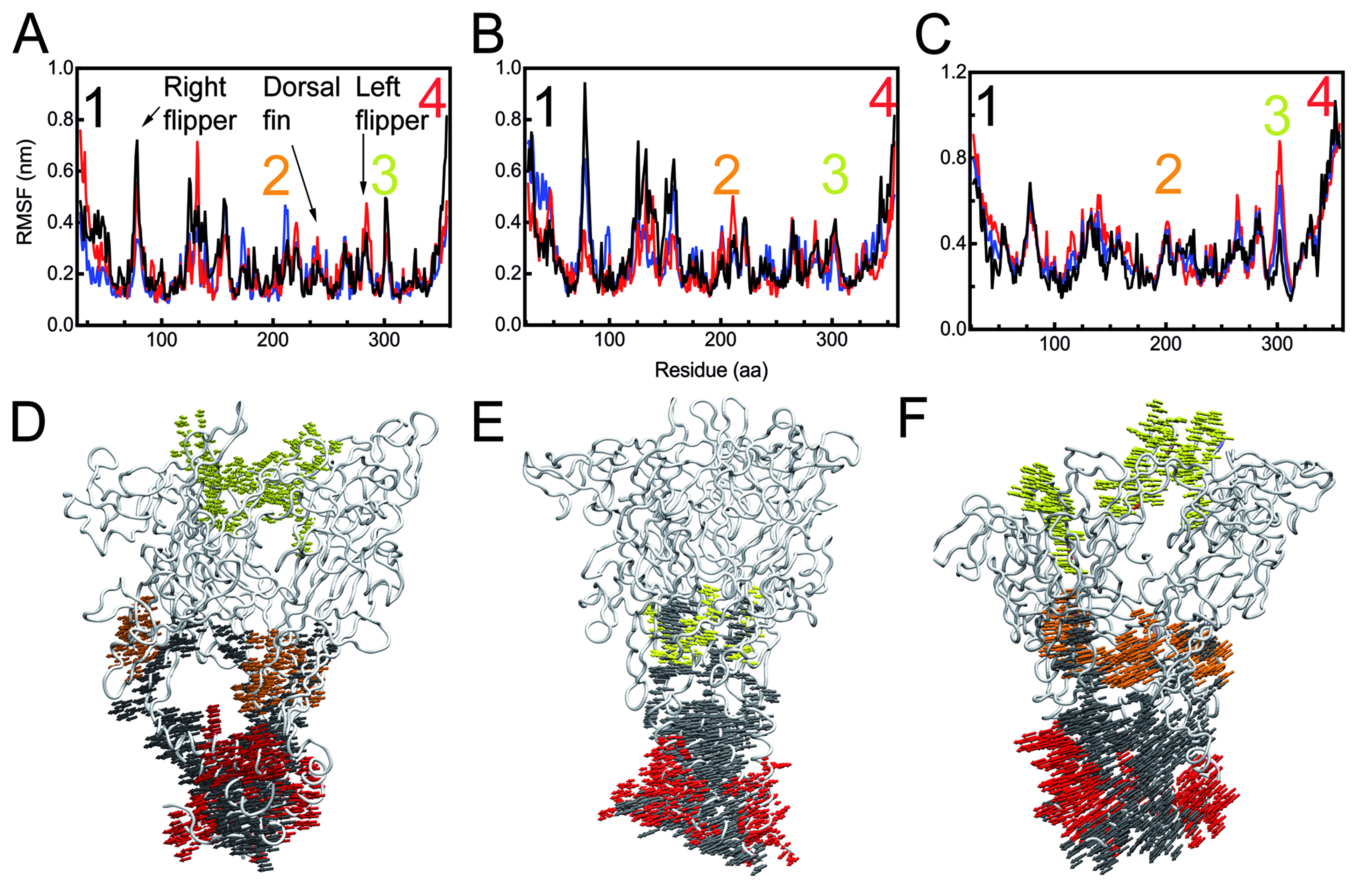

Supplement: Supplementary Figure 3 [file cddiscovery20155-s4.tiff]

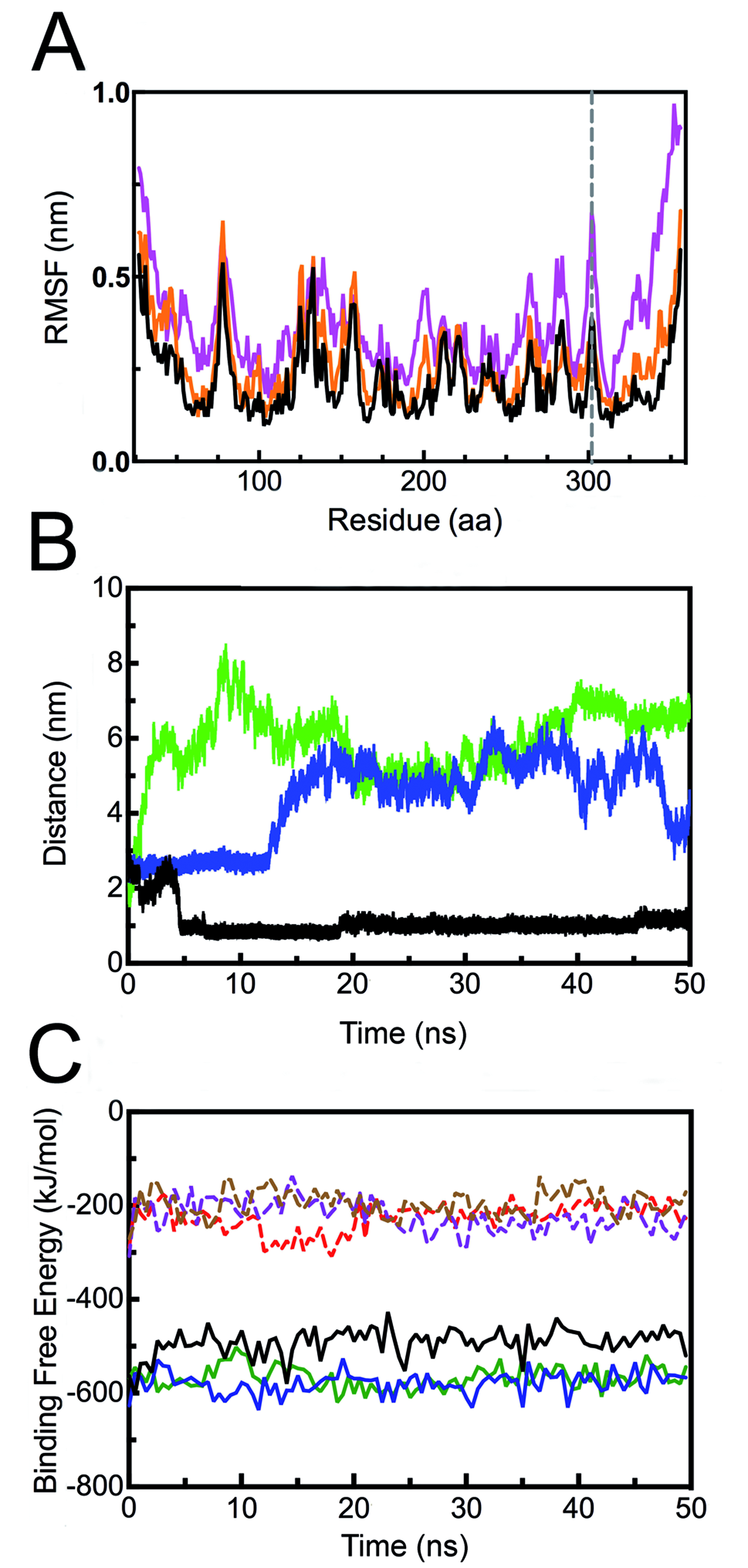

Supplement: Supplementary Figure 4 [file cddiscovery20155-s5.tiff]
