# Supplementary material for: Post-translational allosteric activation of the P2X7 receptor through glycosaminoglycan chains of CD44 proteoglycans
Source: Cell Death Discov. 2015 Oct 5;1:15005–. doi: 10.1038/cddiscovery.2015.5 (PMC4979527; doi:10.1038/cddiscovery.2015.5)
Supplement: Supplementary Table 1 [file cddiscovery20155-s6.doc]

TABLE SI1

Sequences primers used in real time qPCR

| **GENE** | **SEQUENCES PRIMERS** |
| --- | --- |
| Syndecan 1 | Foward: GAC TCT GAC AAC TTC TCT GGC  Reverse: GCT GTG GTG ACT CTG ACT GTT G |
| Syndecan 2 | Foward: CAG GAG CTG ATG AAG ACA TAG AGA  Reverse: ATG AGG AAA ATG GCA AAG AGA A |
| Syndecan 3 | Foward: TCG TTT CCT GAT GAT GAA CTA GAC  Reverse: GTG CTG GAC ATG GAT ACT TTG TT |
| Syndecan 4 | Foward: AGA GCC CAA GGA ACT GGA AGA GAA  Reverse: ATC AGA GCT GCC AAG ACC TCA GTT |
| Glypican 1 | Foward: ACT CCA TGG TGC TCA TCA CTG ACA  Reverse: TTT CCA CAG GCC TGG ATG ACC TTA |
| Glypican 2 | Foward: TCT TTG GCT CAG CTC TTC TCG CAT  Reverse: ACT GTA TTG TGG GTG CAG CAA AGG |
| Glypican 3 | Foward: AAT GAT ACC CTG TGC TGG AAC GGA  Reverse: AGA ACT TAC CCT TGG GCA CAG ACA |
| Glypican 4 | Foward: ACA ACC CAG AAG TCC AGG TTG ACA  Reverse: ACT CCG AAG GGC ACT GCT GAT ATT |
| Glypican 5 | Foward: CAC GTG CTC CTG AAC TTC CAC TTG  Reverse: AAC ACA AAG CTG GTC AGC CAG GCC |
| Glypican 6 | Foward: GTC CGG ACC TAC GGG ATG CTG TAC  Reverse: TCT TCC ATT TCT GTG GTG CAG CAGG |
| CD 44 | Foward: ACC TCT GCC AGG CTT TCA ACA GTA  Reverse: TGT GTC ATA GTG GGA GGT GTT GGA |
| HPRT | Foward: CTC ATG GAC TGA TTA TGG ACA GGA C  Reverse: GCA GGT CAG CAA AGA ACT TAT AGC |
| GAPDH | Foward : AAG AAG GTG GTG AAG CAG GCA TCT  Reverse: ACC CTG TTG CTG TAG CCG TAT TCA |
| Beta-Actin | Foward: ACT CTT CCA GCC TTC CTT C  Reverse: ATC TCC TTC TGC ATC GTG TC |
